# Advances of radiolabeled GRPR ligands for PET/CT imaging of cancers

**DOI:** 10.1186/s40644-024-00658-y

**Published:** 2024-01-26

**Authors:** Yuze Ma, Feng Gao

**Affiliations:** Research Center for Experimental Nuclear Medicine, School of Basic Medical Sciences, Cheeloo College of Medicine, Shandong University, Jinan, 250012 Shandong China

## Abstract

GRPR is a type of seven-transmembrane G-protein coupled receptor that belongs to the bombesin protein receptor family. It is highly expressed in various cancers, including prostate cancer, breast cancer, lung cancer, gastrointestinal cancer, and so on. As a result, molecular imaging studies have been conducted using radiolabeled GRPR ligands for tumor diagnosis, as well as monitoring of recurrence and metastasis. In this paper, we provided a comprehensive overview of relevant literature from the past two decades, with a specific focus on the advancements made in radiolabeled GRPR ligands for imaging prostate cancer and breast cancer.

## GRPR

**Fig. 1:**
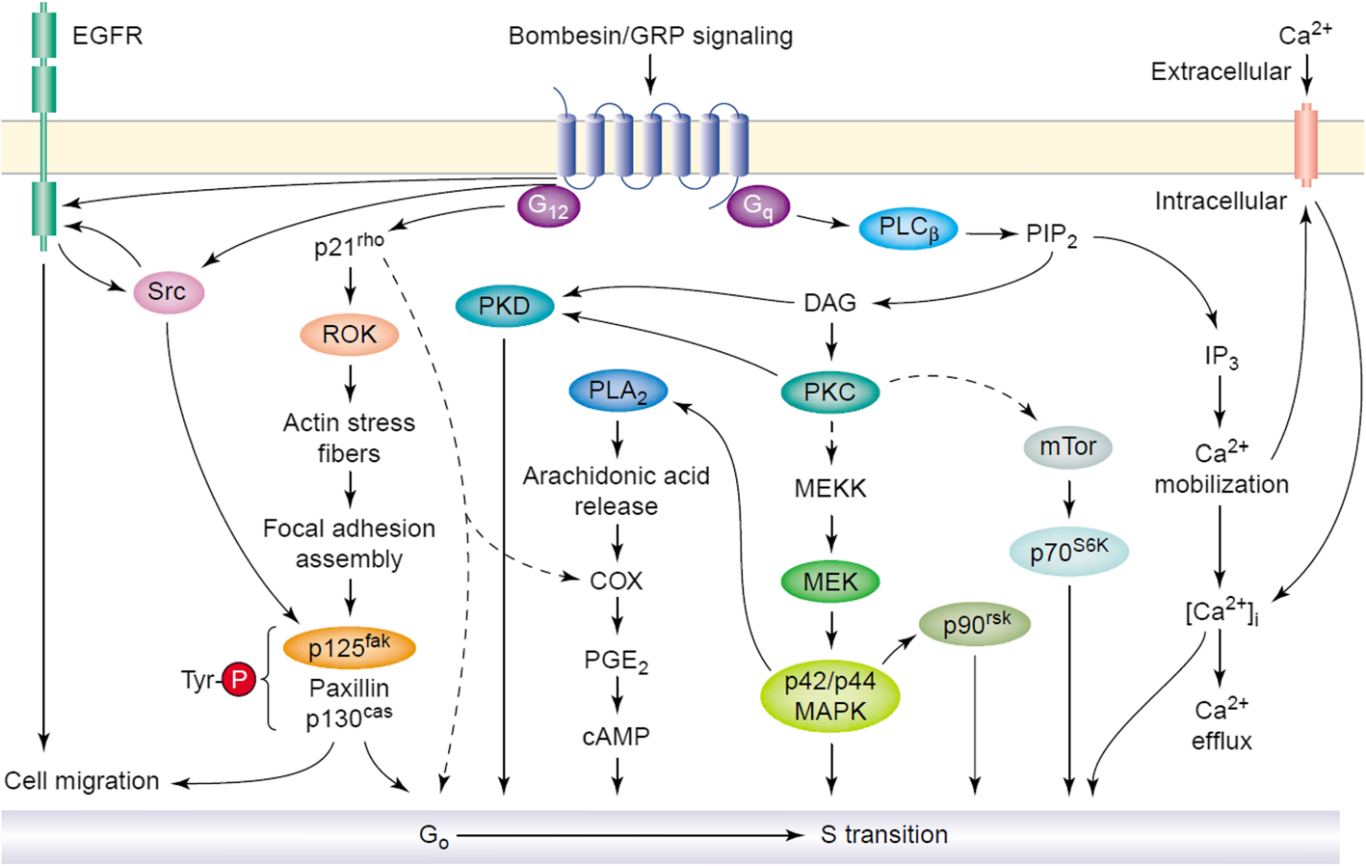
Signal transduction pathways activated by engagement of the bombesin/GRP receptor, a paradigm of mitogenic GPCR. The binding of the ligand (e.g. bombesin) to the cognate GPCR (e.g. the bombesin/GRP preferring receptor) induces activation of the heterotrimeric G proteins of the Gq and G12 subfamilies. Signaling through Gq/G11 leads to PLC activation, hydrolysis of PIP2, generation of IP3 and DAG, and activation of subsequent phosphorylation cascades leading to the activation of ERKs, p70S6K, and PKD. These pathways are representative of studies in Swiss 3T3 fibroblasts, SCLC cell lines, and pancreatic cancer cells. In other cell types, activation of tyrosine phosphorylation pathways including Src, EGFR, and/or Pyk-2 promote Ras-mediated ERK activation via the SOS–Grb2 complex. Signaling through the G12 subfamily (comprising Gα12 and Gα13) transduces GPCR signals into Rho activation, actin remodeling, assembly of focal adhesions, and tyrosine phosphorylation of the focal adhesion-associated proteins FAK, CAS, and paxillin, and complex formation between FAK and Src. These pathways are implicated in both cell proliferation and cell migration. With permission, from Ref. [12]

The Gastrin Releasing Peptide Receptor (GRPR) is a G-protein-coupled receptor that belongs to the bombesin protein receptor family [1]. The natural ligand for GRPR is gastrin releasing peptide (GRP). The C-terminal peptide sequence of GRP shares high similarity with the amphibious peptide bombesin, a 14-amino acid peptide that exhibits high affinity for GRPR and the neuromedin B receptor. Both GRP and bombesin bind strongly to GRPR in the nanomolar range. When these peptides bind to GRPR, they initiate downstream signaling cascades and activate various physiological and biological effects, including cell proliferation, differentiation, and mitosis [2–4]. Currently, all GRPRs are characterized as G-protein-coupled receptors with seven transmembrane domains. The primary signaling cascade involves the activation of phospholipase C (PLC), resulting in intracellular calcium changes, the production of diacylglycerol, and the activation of protein kinase C (PKCs) [2, 5–8]. These effects are primarily achieved through coupling with heterotrimeric G proteins of the Gq/11 and G12/13 families [5, 9, 10]. Other intracellular mediators activated by GRPRs include mitogen-activated protein kinases, adhesion kinases, phosphatidylinositol 3-kinases, and, in certain cases, cyclic AMP response element binding proteins (Fig. 1) [11–13]. GRPR is involved in various physiological mechanisms within the human body. For instance, it regulates gastrointestinal movement and gastric emptying, as well as induces smooth muscle contraction [14]. Endogenous gastrin release is stimulated by activating sensory neurons in the gastric mucosa [15, 16]. Furthermore, GRPR plays a role in the regulation of trypsin release [17] and is involved in immune responses [18, 19]. In addition to these functions, GRPR is implicated in certain brain functions such as the regulation of circadian rhythm [20, 21], memory [22], and the modulation of stress, fear, and anxiety [23–25].

## Expression of GRPR in different cancers

GRPR has garnered significant interest in the fields of oncology and nuclear medicine due to its high-density expression in various cancers, including prostate cancer (PC) [26–28], breast cancer (BC) [29–32], small cell lung cancer (SCLC) [33], gastrinoma, gastrointestinal stromal tumors [34, 35], and other cancers [36]. Notably, apart from the pancreas and gastrointestinal tract, there is minimal physiological expression of GRPR in other tissues [37]. In the following section, we make a concise overview of GRPR expression in several common tumors.

The expression of the GRPR gene was assessed in fresh frozen specimens from 12 cases of PC and 6 cases of benign prostatic hyperplasia using Reverse Transcription-Polymerase Chain Reaction (RT-PCR) and in situ hybridization techniques. The findings revealed that GRPR was expressed in both PC and benign prostatic hyperplasia tissues. The concentration of GRPR mRNA in cancerous tissue varied widely, ranging from very high to undetectable levels (approximately 30% of cases), whereas the concentration of GRPR mRNA in normal tissue remained consistently low [38]. The radioactive ligand ^125^I-Tyr^4^-bombesin was utilized to examine the expression of GRPR, and the positive expression rate of GRPR in invasive PC (30/30) and in prostatic intraepithelial proliferative lesions (26/26) was found 100%. In hyperplastic prostate glands, GRPR was only detected in a few instances, and its density was substantially lower in glandular tissues, primarily localized in certain interstitial tissues [26]. Among the 80 PC specimens tested using ^125^I-Tyr^4^-bombesin receptor binding assays, 63% exhibited high-affinity and low-volume binding sites for bombesin/GRP, with 12 receptor-positive samples displaying two types of binding sites. Concurrently, 91% of PC samples analyzed via RT-PCR expressed GRPR mRNA [39]. GRPRs were also confirmed in various PC cell lines [40, 41]. Furthermore, when assessing GRPR levels in 80 cases of primary PC using receptor binding tests, 68% demonstrated a high affinity to GRPR. Subsequent analysis of these samples through RT-PCR revealed high levels of GRPR mRNA in 91% of cases, while the detectable GRPR mRNA level in non-neoplastic prostate tissue remained significantly low. These observations strongly suggested that GRPR may serve as a molecular marker for precancerous changes in PC [42]. Given the overexpression of GRPR in PC and its high labeling efficiency, GRPR-targeted radioligands have been extensively investigated and applied in PC diagnosis and treatment [27].

GRPR is predominantly expressed in the majority of BCs, while it is not detected in normal breast epithelial cells [36]. Among BC cell lines, GRPR was found in 38% of cases, whereas long-term cultured normal breast epithelial cells did not exhibit GRPRs [43]. In a study conducted by Halmos et al., the binding of ^125^I-Tyr^4^-bombesin to cell membranes isolated from 100 human BC cases revealed that 33% of individuals expressed GRPRs [44]. M. Gugger et al. utilized in vitro receptor autoradiography to evaluate the presence of GRPRs in human breast tissues, including both non-tumor and tumor samples, and found GRPR expression in 63% of cases diagnosed with invasive ductal carcinoma and 65% of cases diagnosed with ductal carcinoma in situ. Notably, the expression of GRPRs was predominantly observed within the neoplastic breast epithelial cells, exhibiting a high density but an uneven distribution [29]. Furthermore, a comprehensive study employing in vitro autoradiography to examine the expression of GRPR subtypes reported that 72% (41 out of 57) of BCs expressed GRPRs [36].

The expression of GRPR in lung cancer has been extensively studied. Several investigations have reported the overexpression of GRPR in non-small cell lung cancer (NSCLC) and its potential role in promoting tumor growth. In a study by Paola et al., the occurrence frequency, relative quantitative expression, activation signaling, and impact on cell growth of GRPR were examined in 13 different human lung cancer cell lines. The findings revealed that GRPR could stimulate the breakdown of inositol phosphate, induce changes in intracellular calcium levels, increase MAPK phosphorylation, promote cell growth, trigger trans-activation of EGFR in specific lung cancer cell lines, and influence cell signaling and growth. Additionally, the activation of GRPR was found to enhance the survival of lung cancer cells exposed to tyrosine kinase inhibitors (TKIs) [36]. Approximately two-thirds of SCLC tumors produce gastrin-releasing peptide precursor (pro-GRP), which establishes the theoretical basis for pro-GRP as a specific tumor marker for SCLC. Subsequent experiments have demonstrated that pro-GRP can not only be utilized in the early diagnosis of SCLC but also aid in assessing treatment effectiveness and detecting tumor recurrence in a timely manner [45]. Furthermore, high expression of GRPR has been observed in several other types of cancer, although further details on these findings are beyond the scope of this discussion.

## PET/CT

Positron emission tomography/computed tomography (PET/CT) represents a novel imaging modality that synergistically combines two advanced techniques: functional metabolic imaging (PET) and anatomical structural imaging (CT). By administering a small amount of positron-emitting radiotracer into the human body and utilizing specialized detectors, PET/CT facilitates the assessment of positron annihilation distribution in various organs. Concurrently, computer tomography enables precise localization of uptake of the PET emitter, thereby offering comprehensive visualization of the physiological and metabolic functions of major human organs. This integration harnesses the respective advantages of PET and CT, maximizing their potential [46, 47]. Considered an advanced imaging technology within the realm of nuclear medicine, PET/CT serves as a powerful tool to capture changes in disease-related physiological functions [48–50]. By merging PET's functional imaging capabilities with CT's anatomical information, PET/CT provides molecular-level insights into tissue cell metabolism, function, blood flow, cell proliferation, and receptor distribution. It has as extensive applications in oncology, cardiovascular diseases, neurology, and other fields, providing vital diagnostic information for physiological and pathological conditions. Consequently, PET/CT has become an indispensable imaging modality in clinical practice [51, 52].

## Application of radionuclide labeled GRPR agonist/antagonist in PET imaging of various cancers

Nowadays, a lot of radionuclide-labeled GRPR agonists/antagonists have been developed and applied in tumor imaging and treatment. Over the past two decades, extensive studies were focused on various types of cancer, with particular emphasis on PC and BC, as these two cancers have the highest incidence rates among men and women, respectively, in western countries. Furthermore, PC and BC are associated with substantial morbidity and mortality, especially during the metastatic stage [53]. Hence, there is a critical need for non-invasive and reliable methods to diagnose and stage these cancers. Biopsies, which are often inconclusive, can cause patient discomfort, anxiety, and increased medical costs [54, 55]. Conventional imaging techniques, including MRI, CT, ultrasound, and even established nuclear medicine procedures like ^18^F-FDG-PET, have limited diagnostic value due to their lack of specificity. Consequently, receptor-targeted imaging has emerged as an appealing alternative for achieving highly specific and sensitive diagnosis of primary and metastatic diseases. The high expression of GRPR in pathological lesions offers great promise for application of receptor-targeted imaging in PC and BC. Other types of cancer are rarely reported or only briefly described.

### Prostate cancer (PC)

The bombesin protein [56–58], an amphibian counterpart of mammalian gastrin-releasing peptide, has been extensively utilized in the development of molecular probes for GRPR imaging. Specifically, the fragment peptide BBN (7–14) has gained significant popularity. Initially, radiolabeled bombesin analogues were created to target GRPR-positive tumors in vivo, primarily due to their rapid and extensive internalization into cancer cells [59, 60]. At that time, internalization was considered crucial for prolonging retention and potentially improving diagnostic sensitivity and therapeutic effectiveness. However, mounting evidence suggests that radiolabeled GRPR antagonists exhibit surprisingly superior capabilities in visualizing GRPR-positive tumors in vivo [61, 62]. Notably, GRPR antagonists also offer an advantage in terms of biological safety. In comparison to agonists, antagonists do not induce pharmacological effects following receptor binding, resulting in better tolerance after intravenous administration.

Rosalba Mansi et al. conducted a comparative analysis between the newly developed ^111^In/^68^Ga-labeled bombesin antagonist RM1 and the GRPR-targeting agonist ^111^In-AMBA. The IC_50_ value of ^nat^In-RM1 was 14 ± 3.4 nM. ^nat/111^In-RM1 was found to bind to the GRPR with a K_d_ of 8.5 ± 2.7 nM compared with a K_d_ of 0.6 ± 0.3 nM of ^111^In-AMBA. A higher B_max_ value was observed for ^111^In-RM1 (2.4 ± 0.2 nM) compared with ^111^In-AMBA (0.7 ± 0.1 nM). Additionally, the researchers investigated the biodistribution and imaging in PC-3 tumor-bearing nude mice. The efficacy of the antagonists was assessed by examining their impact on calcium release and receptor internalization, which were monitored through immunofluorescence microscopy. Despite exhibiting relatively low affinity for GRPR, the antagonist ^111^In/^68^Ga-RM1 demonstrated superior targeting capabilities compared to ^111^In-AMBA. These findings suggested that radiolabeled GRPR antagonists hold greater potential than radiolabeled agonists for in vivo imaging and targeted radiotherapy of GRPR-positive tumors [62].

Rosalba Mansi et al. introduced a novel compound, RM2, labeled with radioactive metals such as ^111^In and ^68^Ga. The researchers synthesized RM2 and conducted in vitro evaluations using PC-3 cells. The IC_50_ values were 7.7 ± 3.3 nM for RM2 and 9.3 ± 3.3 nM for ^nat^In-RM2, The *K*_d_ value for ^111^In-RM2 was 2.9 ± 0.4 nM while the B_max_ value was 1.1 ± 0.05 nM. The efficacy of the antagonists was evaluated through immunofluorescence-based internalization and calcium mobilization tests. Furthermore, the in vivo distribution of ^111^In-RM2 and ^68^Ga-RM2, as well as PET imaging of ^68^Ga-RM2 were performed in mice bearing PC-3 and LNCaP tumors. The findings demonstrated that RM2 and ^111^In-RM2 exhibited high affinity and selectivity as ligands for GRPRs. Immunofluorescence-based internalization and calcium mobilization experiments confirmed the absence of agonist effects. Both ^68^Ga-RM2 and ^111^In-RM2 displayed substantial tumor-specific uptake, particularly in the pancreas. Tumor uptake remained high, while the clearance rate in the pancreas and other abdominal organs was relatively rapid. Pharmacokinetic and imaging studies indicated that ^111^In-RM2 and ^68^Ga-RM2 were suitable candidates for clinical SPECT and PET investigations [63].

The elevated expression of GRPR in PC compared to benign prostatic hyperplasia presents a promising target for PC staging and monitoring. Based on the assumption of increased metabolic activity of cancer cells, metabolic-based tracers are also employed for PC imaging. R.P.J. Schroeder et al. conducted a study comparing GRPR-based targeting using ^68^Ga-labeled bombesin analogue AMBA and metabolism-based targeting using ^18^F-methylcholine (^18^F-FCH) in nude mice implanted with human prostate VCaP xenografts. Uptake was 6.7 ± 1.4%ID/g (*N* = 8) for ^68^Ga-AMBA, and only 1.6 ± 0.5%ID/g (*N* = 8) for ^18^F-FCH. This difference was highly significant (*p* < 0.001). Similarly, for PC-3 tumors, uptake was 9.2 ± 1.1%ID/g (*N* = 3) for ^68^ Ga-AMBA and 1.2 ± 0.3%ID/g (*N* = 3) for ^18^F-FCH. Dynamic PET images were reconstructed and quantitatively analyzed. The study revealed that ^68^Ga-AMBA effectively visualized all tumors, whereas ^18^F-FCH exhibited significantly lower contrast due to its inferior tumor-to-background ratio. PET quantitative analysis demonstrated rapid tumor uptake and high retention rates for both tracers. Similar results were observed in PC-3 tumor-bearing mice. The biodistribution data aligned with the PET findings, showing higher tumor uptake of ^68^Ga-AMBA compared to ^18^F-FCH in VCaP tumors. Apart from GRPR-expressing organs, the uptake of ^68^Ga-AMBA in other organs was lower than that of ^18^F-FCH. In the same PC tumor-bearing mice, the tumor uptake of ^68^Ga-AMBA was higher than that of ^18^F-FCH, while the overall background activity was lower. These results suggested that peptide receptor-based targeting using the bombesin analogue AMBA was superior to choline-based metabolic targeting in radionuclide imaging of PC [64].

**Fig. 2:**
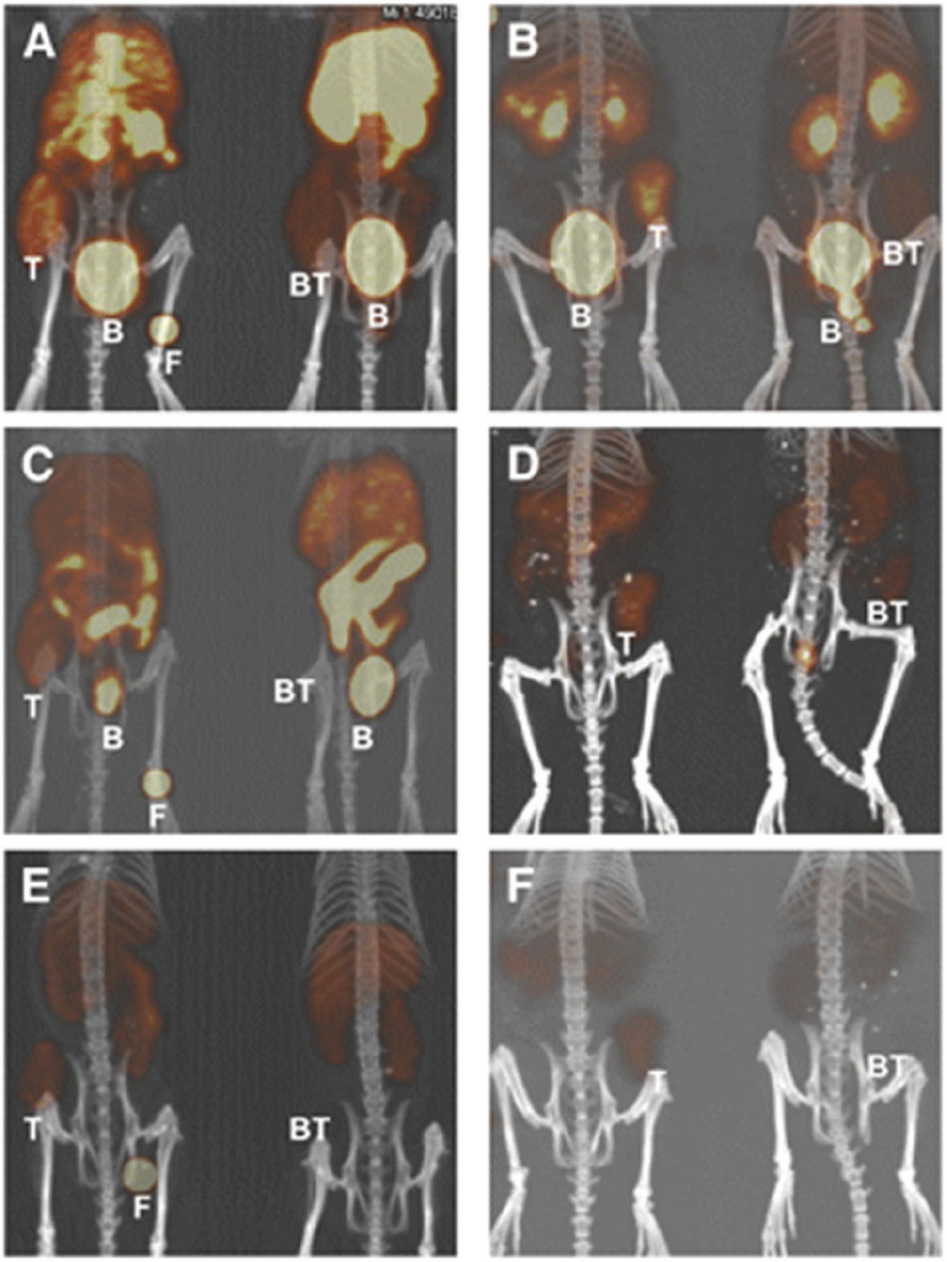
Coronal views of maximum-intensity projections of small-animal PET images with coregistered CT image of mice bearing PC-3 xenografts in rear flank at 1 (**A** and **B**), 4 (**C** and **D**), and 24 h (**E** and **F**). Mice were injected intravenously with ^64^Cu-SarAr-SA-Aoc bombesin(7–14) (**A**, **C**, and **E**; tumors on the left flank) and ^64^Cu-SarAr-SA-Aoc-GSG bombesin(7–14) (**B**, **D**, and **F**; tumors on the right flank). Mice on the left of each frame were not injected with a blocking agent, whereas mice on right received 100 µg of Tyr^4^-bombesin as an inhibitor. Fiducial markers (**F**) are also shown in some images. B = bladder; BT = blocked tumors; T = nonblocked tumors. *This* *study* *was originally published in JNM. Lears, K.A., et al., In vitro and in vivo evaluation of 64Cu-labeled SarAr-bombesin analogs in gastrin-releasing peptide receptor-expressing prostate cancer.* *J Nucl Med, 2011. 52(3): p. 470–7. © SNMMI*

Several studies have utilized different chelating agents to label the C-terminal eight amino acids of bombesin (7–14) with ^64^Cu. These analogues have demonstrated GRPR-specific PET imaging capabilities in small animal tumor models, but they came with various advantages and disadvantages. ^64^Cu-labeled compounds may be superior to ^68^Ga-labeled compounds in the future because of the longer half-life of ^64^Cu (12.7 h), which would allow for a longer time span for PET imaging. In the research conducted by Kimberly A. Lears et al., a chelating agent called SarAr was employed to conjugate with bombesin. They synthesized SarAr and conjugated SarAr with bombesin(7–14) via solid-phase synthesis method, obtaining SarAr-SA-Aoc-bombesin(7–14) and SarAr-SA-Aoc-GSG-bombesin(7–14). A competitive binding assay was performed using PC-3 cells and ^125^I-Tyr^4^-bombesin to determine the half-maximal inhibitory concentration (IC_50_). These peptide conjugates were labeled with ^64^Cu, and their internalization in PC-3 cells in vitro and the uptake in PC-3 xenografts in mice were evaluated. The results of the competitive binding assay indicated that both SarAr-SA-Aoc-bombesin(7–14) and SarAr-SA-Aoc-GSG-bombesin(7–14) exhibited high affinity for GRPR, with IC_50_ values of 3.5 nM and 4.5 nM, respectively. Both peptides were successfully labeled with ^64^Cu and displayed similar levels of internalization in PC-3 cells. In vivo, these radiolabeled peptides demonstrated tumor-specific uptake and exhibited improved imaging performance compared to previously reported ^64^Cu-labeled bombesin analogues. ^64^Cu-SarAr-Aoc-GSG-bombesin(7–14) exhibited faster blood clearance and lower uptake in tumor and normal tissues compared to ^64^Cu-SarAr-SA-Aoc-bombesin(7–14), resulting in similar tumor-to-blood ratios for both analogues. Both ^64^Cu-SarAr-Aoc-bombesin(7–14) and ^64^Cu-SarAr-SA-Aoc-GSG-bombesin(7–14) possessed high affinity for GRPR-expressing cells and had potential for PC PET imaging (Fig. 2) [65].

Chatalic K.L. et al. conducted a comparative study on three novel GRPR-targeting tracers: Al^18^F-JMV5132, ^68^Ga-JMV5132, and ^68^Ga-JMV4168. GRPR antagonist JMV594 [66] was coupled with NODA-MPAA, and labeled with Al^18^F. JMV5132 was labeled with both ^68^Ga and ^18^F, while JMV4168 was labeled with ^68^Ga. The inhibitory concentration of JMV4168, JMV5132, ^nat^Ga-JMV4168, ^nat^Ga-JMV5132 and Al^nat^F-JMV5132 to GRPR were determined using a competitive binding assay. IC_50_ values were 6.8 nM for JMV5132, 13.2 nM for JMV4168, 3.0 nM for ^nat^Ga-JMV5132, 3.2 nM for ^nat^Ga-JMV4168, and 10.0 nM for Al^nat^F-JMV5132. The tumor targeting ability of these compounds was evaluated in mice with subcutaneously transplanted PC-3 tumors. Small animal PET/CT images were acquired, and the biodistribution of the tracers was determined through in vitro measurements. The study revealed that Al^18^F-JMV5132 could be accomplished within 20 min. In mice with PC-3 tumors, all tracers exhibited rapid clearance from the blood. ^68^Ga-JMV4168 was predominantly cleared via the kidneys, while ^68^Ga-JMV5132 and Al^18^F-JMV5132 showed partial clearance from the liver and gallbladder. Small animal PET/CT imaging clearly visualized PC-3 tumors, with Al^18^F-JMV5132 displaying the highest resolution. The study demonstrated that Al^18^F-JMV5132, ^68^Ga-JMV5132, and ^68^Ga-JMV4168 exhibited specific accumulation in GRPR-positive PC-3 tumors. These novel PET tracers hold promise as potential candidates for future clinical imaging applications [67].

Theodosia Maina et al. introduced ^68^Ga-SB3, as an alternative to ^99m^Tc-labeled tetraamine using the chelating agent DOTA for PET imaging with the radioactive metal ^68^Ga. The researchers conducted competitive binding experiments of SB3 and ^nat^Ga-SB3 using [^125^I-Tyr^4^] BBN on PC-3 cell membranes. Blood samples were collected from mice after injecting the ^67^Ga-SB3 surrogate, and the degradation products were analyzed using high-performance liquid chromatography (HPLC). Biodistribution studies were performed in severe combined immunodeficient mice with PC-3 xenograft tumors after injecting ^67^Ga-SB3. Additionally, 17 patients with BC (8 cases) and PC (9 cases) were injected with ^68^Ga-SB3 and PET/CT fusion images were obtained. The results demonstrated that SB3 and ^nat^Ga-SB3 exhibited high affinity for human GRPR, and ^67^Ga-SB3 showed good in vivo stability. ^67^Ga-SB3 displayed higher retention in PC-3 xenografts but faster clearance from the GRPR-rich pancreas. Among the patients, no adverse reactions were observed following administration of ^68^Ga-SB3, and 50% of the BC patients (4 out of 8) and 55% of the PC patients (5 out of 9) exhibited pathological uptake of ^68^Ga-SB3 on PET/CT imaging. In PC-3 tumor-bearing mice, ^67^Ga-SB3 demonstrated favorable pharmacokinetics, while ^68^Ga-SB3 PET/CT imaging revealed approximately 50% of lesions in patients with advanced PC and BC. The researchers anticipated that ^68^Ga-SB3 might provide even better outcomes for patients with BC or PC [68].

In order to investigate the safety and efficacy of ^68^Ga-labeled GRPR antagonist SB3 in PET/CT imaging of primary PC, Ingrid L. Bakker et al. conducted a study focusing on the biological distribution, dosimetry, pathology, and GRPR expression. The study included 10 PC patients scheduled for prostatectomy, who underwent PET/CT imaging within 2 weeks prior to the surgery. The tumor location and Gleason score of prostate tissue were evaluated, and GRPR expression was determined through in vitro autoradiography. The findings demonstrated that ^68^Ga-SB3 was well tolerated, with no significant changes in vital signs and laboratory results. PET/CT imaging using ^68^Ga-SB3 revealed lesions in 8 out of 10 cases. Pathological analysis identified a total of 16 tumor lesions, of which 14 were detected by PET/CT, resulting in a sensitivity of 88%. Additionally, ^68^Ga-SB3 PET/CT imaging showed two large prostate intraepithelial neoplasia uptakes, with a specificity of 88%. Autoradiography of tumor lesions displayed varied expression levels of GRPR, with 4 cases showing negative expression. Notably, two patients who tested negative on PET/CT were found to have GRPR-negative tumors. Among autoradiography-positive tumors, the level of GRPR expression correlated significantly with the uptake of the tracer on PET/CT. Regarding dosimetry, the highest absorbed dose was observed in the pancreas, which exhibited physiological GRPR expression. Following were the bladder wall and kidneys. Based on these results, the researchers concluded that ^68^Ga-SB3 PET/CT was a safe and promising imaging method for early detection of PC [69].

**Fig. 3:**
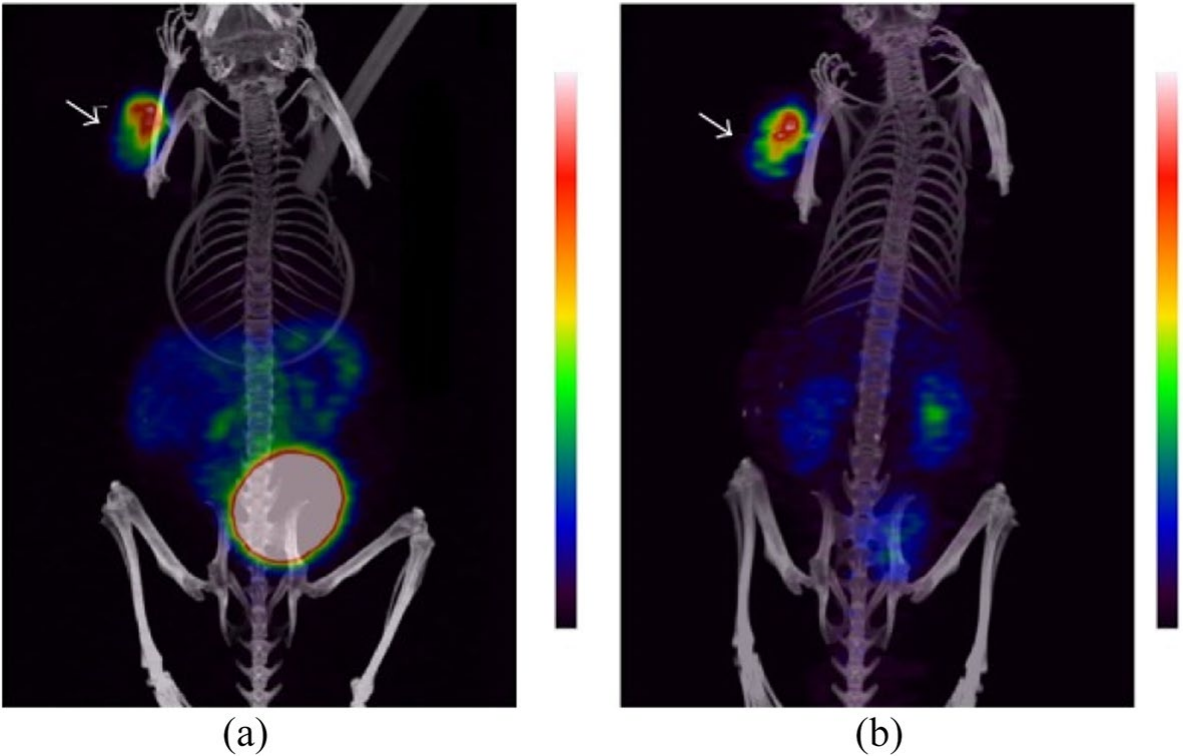
Coronal MIP preclinical PET/CT images showing tracer distribution in PC-3 xenografted NOD-SCID mice. The animals were injected with 0.18 nmol of ^55^Co-NOTA-PEG2-RM26 (approx. 3 MBq) and scanned at (**a**) 3 h and (**b**) 24 h pi. The tumor is shown by the arrow. With permission, from Ref. [70]

Bogdan Mitran et al. have developed a PET imaging agent using ^55^Co-labeled RM26, a GRPR antagonist, to visualize tumors expressing GRPR. They found that the tumor-to-background ratio increased significantly over time to 24 h after injection, due to high uptake and long-term retention in the tumor, as well as rapid clearance from the blood and organs that express GRPR, highlighting the importance of radionuclide half-life in highly sensitive molecular imaging. ^55^Co-NOTA-AMBA provided better imaging contrast than its ^68^Ga-labeled counterpart because it can be imaged at 24 h after injection. Second-day imaging with long-lived radionuclides could detect lower abdominal lymph node involvement, which required the highest possible sensitivity and was the ultimate goal of PC imaging. For second-day imaging, positron-emitting metals with a half-life of 10–20 h are the best choice. Positron-emitting nuclides that may be used for this purpose include ^64^Cu (T_1/2_ = 12.7 h), ^86^Y (T_1/2_ = 14.7 h) and ^55^Co (T_1/2_ = 17.5 h). Among them, the positron abundance of ^55^Co (76% β^+^) is higher than that of ^64^Cu, and the ratio of annihilation photons to co-emitted gamma is higher than ^86^Y, which provides better image quality. The favorable biodistribution profile of Co-labeled NOTA-PEG2-RM26 enabled its use in high-contrast preclinical PET/CT imaging (using ^55^Co) and SPECT/CT imaging (using ^57^Co) (Fig. 3) [70].

**Fig. 4:**
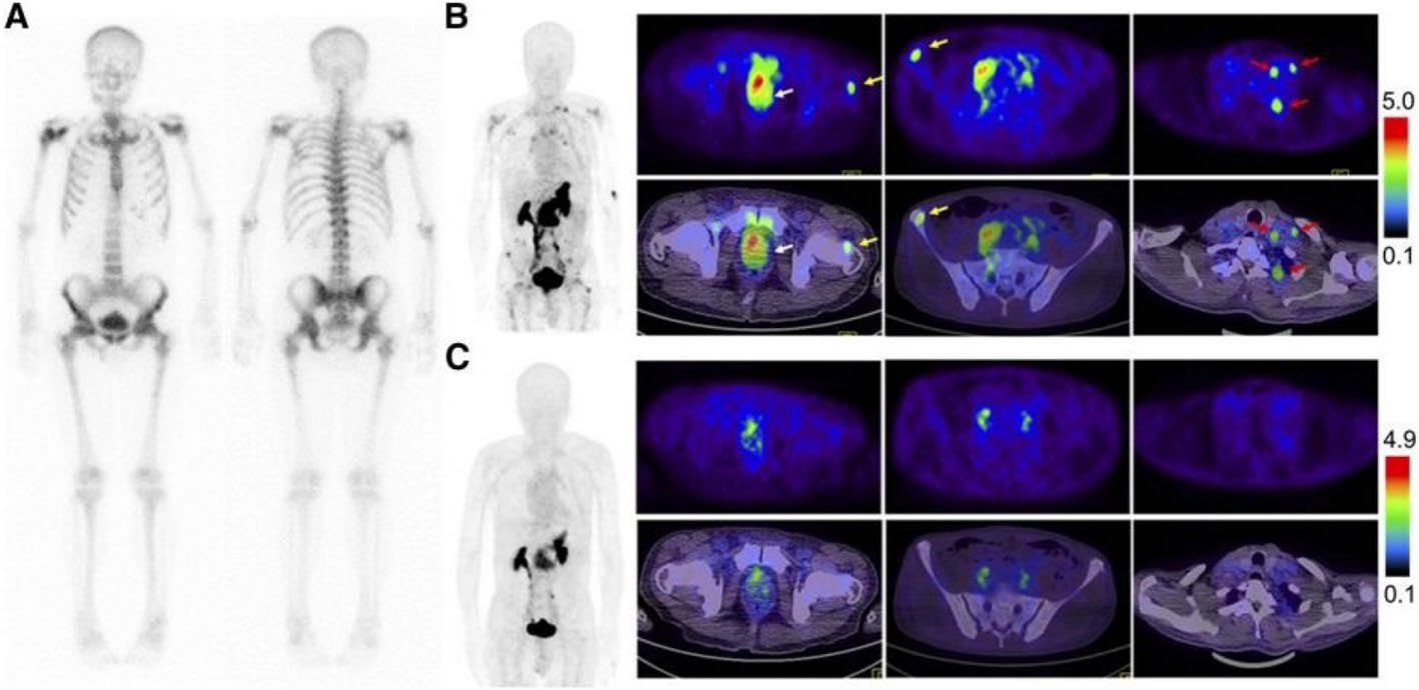
Comparison of ^99m^Tc-MDP bone scintigraphy (**A**), ^68^Ga-RM26 PET/CT (**B**), and ^68^Ga-BBN PET/CT (**C**) in a 73-y-old man diagnosed as having PC (white arrow) with lymph node involvement (red arrow) and bone metastasis (yellow arrow) before prostatectomy. ^68^Ga-RM26 PET/CT detected primary tumors, multiple lymph node involvement, and bone metastasis lesion, whereas those lesions did not significantly show up on ^99m^Tc-MDP bone scintigraphy and showed extremely mild uptake on ^68^ Ga-BBN PET/CT. *This* *study was originally published in JNM*. *Zhang, J., et al.,* *PET Using a GRPR Antagonist (68)Ga-RM26 in Healthy Volunteers and Prostate Cancer Patients. J Nucl Med, 2018. 59(6): p. 922–928. © SNMMI*

Zhang et al. conducted a study to evaluate the safety, biodistribution, radiation dosimetry, and clinical diagnostic value of the GRPR antagonist PET tracer ^68^Ga-RM26 in patients with PC. The study also compared ^68^Ga-RM26 with the GRPR agonist ^68^Ga-BBN. Safety verification and dosimetry calculations of ^68^Ga-RM26 were performed in 5 healthy volunteers. A total of 28 PC patients (17 newly diagnosed and 11 after treatment) participated in the study and provided written informed consent. PET/CT scans were conducted on all cancer patients for 15–30 min following intravenous injection of ^68^Ga-RM26. Among them, 22 patients (11 newly diagnosed and 11 after treatment) were also examined using ^68^Ga-BBN PET/CT within one week as a control. Additionally, ^99m^Tc-MDP bone scans were performed as another control after 2 weeks. Tumor specimens were subjected to GRPR immunohistochemical staining. The results showed that all subjects tolerated ^68^Ga-RM26 well, with no reported adverse symptoms during the procedure or during the 2-week follow-up. In 17 newly diagnosed PC patients, ^68^Ga-RM26 PET/CT showed 15 positive tumors. Among the 11 patients who underwent prostatectomy or brachytherapy, ^68^Ga-RM26 PET/CT detected 8 metastatic lymph nodes in 3 cases and 21 bone metastases in 8 cases. Compared to ^68^Ga-RM26 PET/CT, GRPR agonist ^68^Ga-BBN PET/CT detected fewer primary lesions, lymph node metastases, and showed lower tracer accumulation. The study demonstrated that ^68^Ga-RM26, a GRPR antagonist, was safe and effective. ^68^Ga-RM26 PET/CT hold great value in the diagnosis of PC and PC metastasis. Furthermore, ^68^Ga-RM26 outperforms GRPR agonist as an imaging tracer for evaluating GRPR expression in PC (Fig. 4) [71].

The high pancreatic uptake observed with GRPR targeted radiopharmaceuticals, particularly in targeted radioligand therapy, has been a significant challenge. Wang et al. conducted a study to address this issue by exploring the complex of TacsBOMB2, TacsBOMB3, TacsBOMB4, TacsBOMB5, and TacsBOMB6 derived from the effective GRPR antagonist sequence [Leu^13^GRPRThz^14^]bombesin(7–14), and compared them with ^68^Ga-RM2. PET imaging of PC-3 transplanted tumors demonstrated that ^68^Ga-TacsBOMB2, ^68^Ga-TacsBOMB3, ^68^Ga-TacsBOMB5, ^68^Ga-TacsBOMB6, and ^68^Ga-RM2 exhibited clear visualization of the tumors, with ^68^Ga-TacsBOMB5 displaying the highest tumor uptake rate. Importantly, it was observed that the pancreatic uptake of ^68^Ga-TacsBOMB2, ^68^Ga-TacsBOMB3, ^68^Ga-TacsBOMB5, and ^68^Ga-TacsBOMB6 was significantly lower than that of ^68^Ga-RM2. Among the tested derivatives of ^68^Ga-bombesin(7–14), ^68^Ga-TacsBOMB5 demonstrated the highest tumor uptake rate and the greatest contrast between the tumor and background, indicating its potential for clinical imaging of tumors expressing GRPR [72].

Based on the results of a single-center phase II clinical study by Schollhammer et al., the significant potential of the combination of ^68^Ga-RM2 PET/CT and ^68^Ga-PSMA-617 PET/CT in evaluating different aspects of PC biology was compared. ^68^Ga-PSMA-617 PET/CT is helpful to show the lesions with high ISUP (International Society of Urological Pathology) score and great clinical significance. When the score of ISUP was low, the uptake rate of ^68^Ga-RM2 was higher than that of ^68^Ga-PSMA-617. However, when the score of ISUP was higher, the uptake was similar to that of ^68^Ga-PSMA-617. Importantly, nearly 20% of lesions can only be seen on GRPR- PET (13%) or PSMA (prostate-specific membrane antigen)-PET (6%), which revealed the complementarity of these imaging procedures. The combination of PSMA-PET and GRPR-PET can better classify the lesions in the prostate [73], Compared with PSMA, GRPR has high sensitivity and specificity in patients with prostate cancer. GRPR can play an important complementary role in PSMA-negative tumors and tumors with heterogeneous expression of cell surface receptors. These GRPR ligands have shown reliable detection of various types of PC in patients, representing significant progress in the clinical diagnosis of PC. Here, we list a table (see Table at the end of this article) that briefly summarizes radiopharmaceuticals that target GRPR in PC.

### Breast cancer (BC)

Mammography is a well-established technique for primary BC detection, but it has certain limitations (even under the best conditions of photography and diagnosis, about 5% of BC is false negative due to various reasons. Another major limitation of breast X-ray examination is the differentiation of benign and malignant lesions, which is the same as other system lesions. Breast lesions also have the problem of "different shadow of the same disease, different diseases with the same shadow") that can be addressed through the use of nuclear imaging. Currently available radiopharmaceuticals have limited sensitivity in detecting small primary lesions, necessitating the development of new radiopharmaceuticals for improved detection of primary BC, metastasis, recurrence, and treatment monitoring. As the studies on GRPR progresses, scientists are gradually advancing the application of radionuclide-labeled GRPR agonists/antagonists for the diagnosis and treatment of BC.

In a study conducted by Jesse J. Parry et al., the potential of PET imaging using ^64^Cu-labeled BN analogues was assessed for BC. The binding and internalization of a series of BN analogues, containing linkers with varying carbon lengths (4, 5, 6, 8, and 12), were evaluated in T-47D human BC cells. Subsequently, tissue biodistribution and micro PET imaging were employed to evaluate the performance of ^64^Cu-labeled analogues in mice with T-47D xenografts. The results demonstrated that all analogues exhibited IC_50_ values below 100 nM and were effectively internalized into T-47D cells. Biodistribution studies revealed that BN analogues with 8-carbon connectors exhibited both the highest tumor uptake rate and increased uptake in normal liver tissue. Analogues with 6- or 8-carbon connectors demonstrated favorable tumor uptake, which was further confirmed by micro PET imaging. These findings established the feasibility of utilizing radiolabeled BN analogues for PET detection of GRPR-expressing BC [74].

**Fig. 5:**
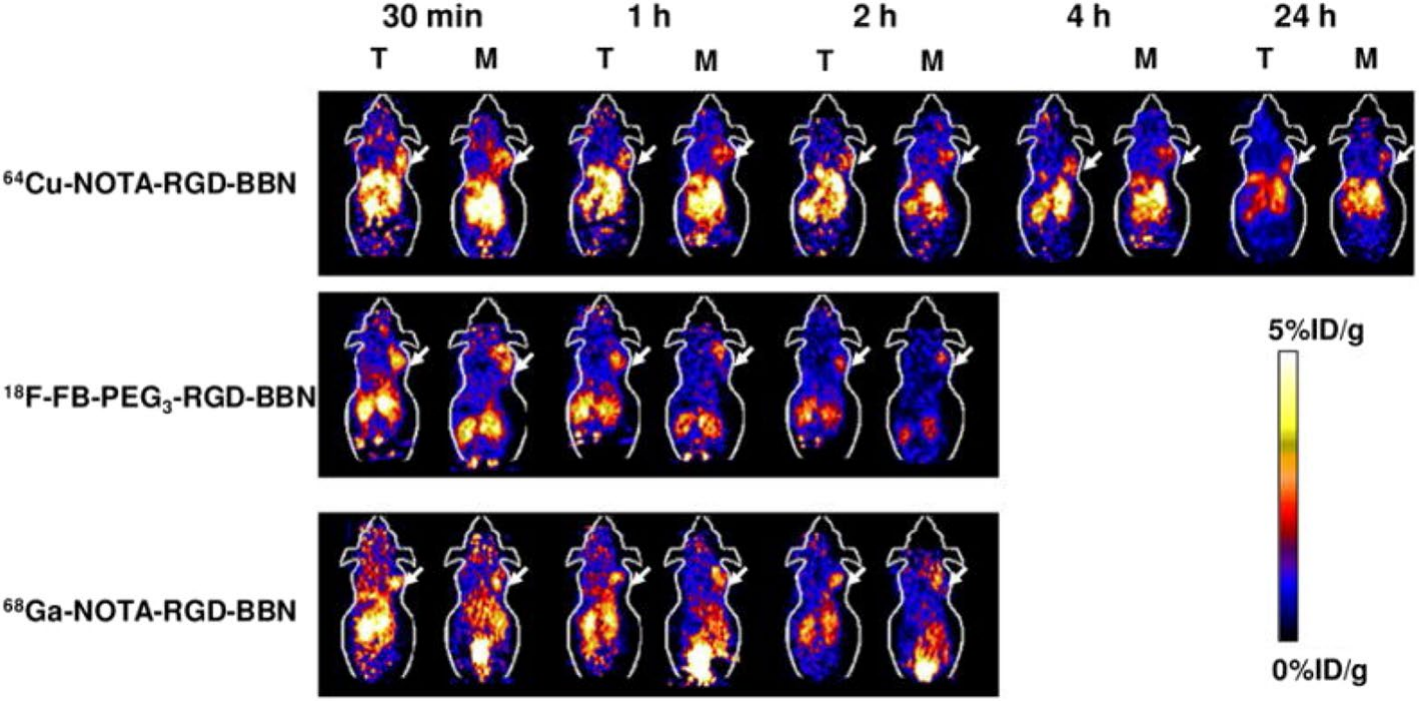
Decay-corrected whole-body coronal microPET images of T47D (T) and MDA-MB-435 (M) tumor-bearing mice at 30 min, 1 h, 2 h, 4 h, and 24 h after injecting 3.7 ~ 5.5 MBq (100 ~ 150 μCi) of ^64^Cu-NOTA-RGD-BBN, ^18^F-FB-PEG3-RGD-BBN or ^68^Ga-NOTA-RGD-BBN. Images shown are static scans of a single mouse, which is representative of the 4 mice tested in each group. Arrows indicate the presence of T47D (T) or MDA-MB-435 (M) tumors. *Reprinted (adapted) with permission from {Liu, Z., et* *al.,* *(18)F, (64)Cu, and (68)Ga labeled RGD-bombesin heterodimeric peptides for PET imaging of breast cancer. Bioconjug Chem, 2009. 20(5): p. 1016–25.}. Copyright {2009} American Chemical* *Society*

RGD and bombesin have shown promise as tumor imaging agents, targeting integrin α_v_β_3_ and GRPR, respectively. In a study by Liu et al., a novel RGD-BBN heterodimer peptide was designed and synthesized, incorporating both RGD and BBN motifs into a single molecule. ^18^F-labeled RGD-BBN heterodimer demonstrated dual targeting capabilities for integrin α_v_β_3_ and GRPR in a PC model (PC-3). The researchers also explored the potential of radiolabeled RGD-BBN tracers for BC detection using micro PET imaging. Cell binding analysis revealed that BC cells expressing high levels of GRPR typically exhibited low to moderate levels of integrin α_v_β_3_, whereas those with high integrin α_v_β_3_ expression displayed minimal GRPR expression. RGD-BBN heterodimers were labeled with three positron-emitting radionuclides, namely ^18^F, ^64^Cu, and ^68^Ga, and the PET studies with these three radiotracers were conducted in T-47D (GRPR^+^/low integrin α_v_β_3_) and MDA-MB-435 (GRPR^−^/integrin α_v_β_3_^+^) BC models. The results demonstrated that all three radiotracers exhibited dual binding affinity for integrin α_v_β_3_ and GRPR in vitro. In MDA-MB-435 tumors models (GRPR^−^/integrin α_v_β_3_^+^), the RGD-BBN radiotracer displayed notable advantages compared to the corresponding BBN analogues. Even though ^18^F-FB-PEG3-RGD-BBN exhibited lower tumor uptake than ^64^Cu-NOTA-RGD-BBN and ^68^Ga-NOTA-RGD-BBN, it provided enhanced contrast for BC visualization. ^64^Cu-NOTA-RGD-BBN exhibited longer tumor retention along with higher liver and kidney uptake, while ^68^Ga-NOTA-RGD-BBN displayed higher tumor uptake but also increased background accumulation. Overall, the labeling groups, chelating agents, and isotopes exerted profound effects on tumor targeting and in vivo kinetics of RGD-BBN tracers that simultaneously recognize dual integrin α_v_β_3_ and GRPR. Further development of radiolabeled RGD-BBN tracers for PET imaging of tumors is warranted (Fig. 5) [75].

In a study conducted by Christophe Van de Wiele et al., immunohistochemistry (IHC) was employed to investigate the uptake of ^99m^Tc-RP527 and its association with GRPR expression in human BC. Nine patients with clinically diagnosed BC and 5 patients with tamoxifen-resistant metastatic BC underwent SPECT imaging using ^99m^Tc-RP527. The results were compared with routine staging examinations of all patients, as well as routine histology and IHC staining in the initial 9 patients. All 9 patients with suspected breast lesions displayed positive tumor uptake. Among these patients, 8 exhibited significant uptake of ^99m^Tc-RP527 in the primary lesions, involved lymph nodes, and certain distant metastases. Conversely, no uptake of ^99m^Tc-RP527 was observed in the tamoxifen-resistant patients. These findings indicated that ^99m^Tc-RP527 possessed a high affinity and binding specificity for primary BC. However, due to the limited samples, further experiments are necessary to establish the clinical significance and potential application of radiolabeled GRPR antagonists in BC imaging [76].

**Fig. 6:**
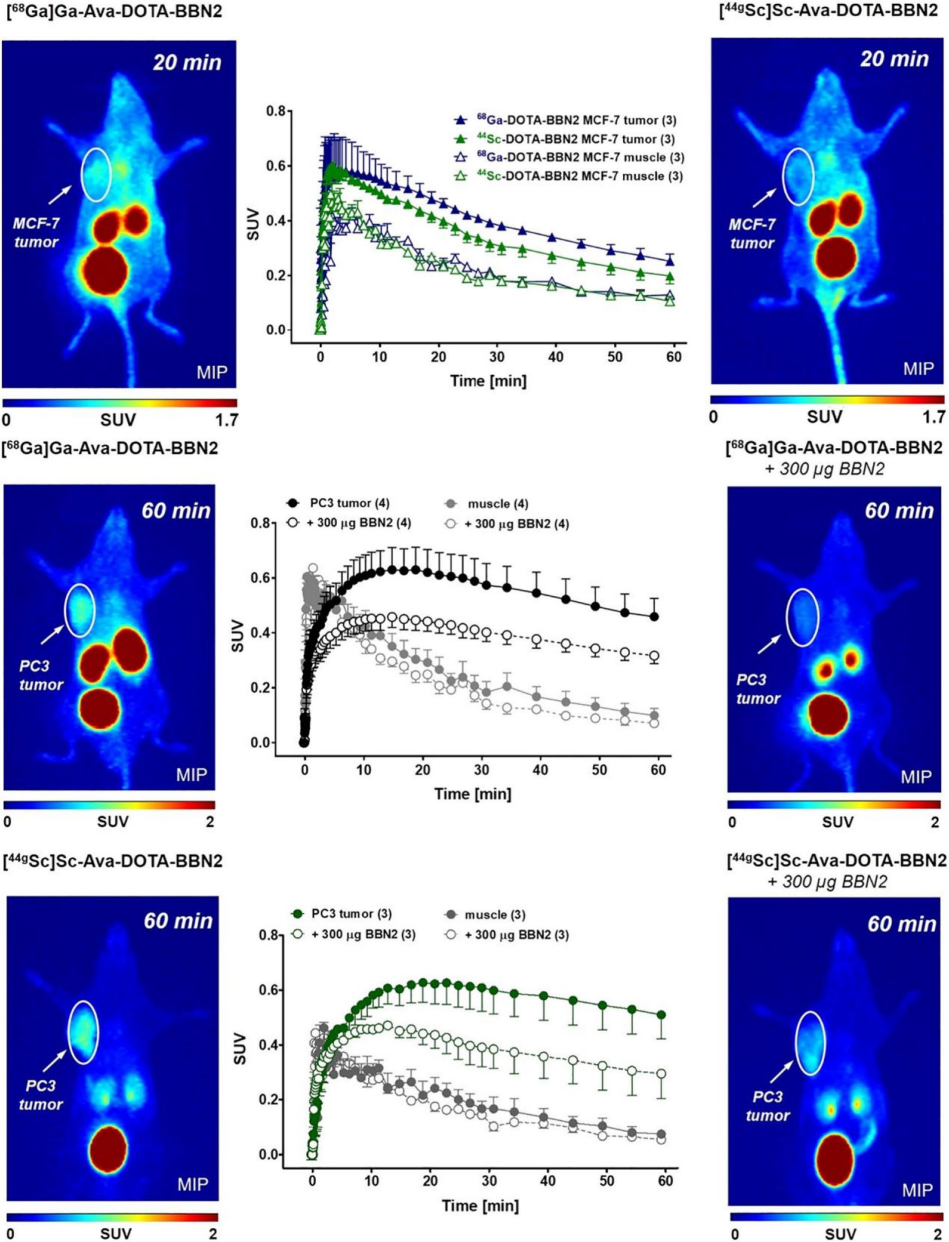
Representative PET images (MIP) of MCF7 tumor-bearing mice at 20 min after injection of ^68^ Ga-DOTA-Ava-BBN2 (left) and ^44g^Sc-DOTA-Ava-BBN2 (right). Corresponding time-activity curves (middle) show the radioactivity levels in the tumor and muscle for both radiopeptides over time as SUV values (mean ± SEM from *n* = 3 experiments). Representative PET images (MIP) of PC-3 tumor-bearing mice at 60 min after injection of ^68^ Ga-DOTA-Ava-BBN2 (top) and ^44g^Sc-DOTA-Ava-BBN2 (bottom) under control (right) and blocking conditions (left). Corresponding time-activity curves (middle) show the radioactivity levels in the tumor and muscle for both radiopeptides over time as SUV values (mean ± SEM from *n* = 3 experiments). With permission, from Ref. [77]

Radiolabeled peptides play a crucial role in targeted imaging and therapy of tumors. In a recent study by Simon Ferguson et al., the metabolically stable GRPR antagonist BBN2 was proposed for labeling with ^18^F and ^68^Ga, enabling PET imaging of GRPR in PC. They focused on the impact of combining ^44g^Sc and ^68^ Ga-labeled DOTA complexes with the GRPR antagonist BBN2 on GRPR affinity in vitro, as well as their biodistribution and tumor uptake in MCF7 and PC-3 models. The DOTA-Ava-BBN2 peptide was labeled with the radionuclides ^68^Ga and ^44g^Sc. The GRPR affinity was assessed in PC-3 cells, and the expression profile of GRPR was studied in human BC tissue samples and MCF7 cells. PET imaging was performed using the ^68^Ga and ^44g^Sc-labeled peptides in xenograft models of MCF7 and PC-3 tumors (Fig. 6). The results demonstrated a high binding affinity for both ^68^Ga-DOTA-Ava-BBN2 and ^44g^Sc-DOTA-Ava-BBN2. Gene expression microarray analysis revealed higher expression of GRPR mRNA in estrogen receptor (ER)-positive BC tissues, which was confirmed by western blotting and immunohistochemistry. However, PET imaging showed lower uptake of these two tracers in MCF7 tumors, while higher tumor uptake and retention were observed in PC-3 tumors models Comparing the biological distribution of DOTA-Ava-BBN2 peptides labeled with ^68^Ga and ^44g^Sc, no differences were observed in MCF7 and PC-3 xenografts in vivo. Both tumor models exhibited similar patterns of tumor uptake and retention, as well as rapid clearance from the blood and kidneys [77].

Christian Stoykow et al. investigated the application of ^68^Ga-RM2 for PET imaging of breast cancer. Prior to ^68^Ga-RM2 PET/CT staging, 15 female BC patients with confirmed biopsies were included. A significant increase in ^68^ Ga-RM2 uptake was observed in 13 out of 18 tumor tissues compared to normal breast tissue (defined as PET positive). All PET-positive primary tumors were found to be ER- and PR-positive (13 out of 13), whereas only one PET-negative tumor was ER- and PR-positive. Normal breast tissue exhibited moderate inter- and intra-individual variability in GRPR binding, while physiological uptake in other organs, except the pancreas, showed a significant decrease. The uptake of ^68^Ga-RM2 in BC was associated with the expression of ER, PR, HER2/neu status, and the MIB-1 proliferation index. Importantly, ^68^Ga-RM2 PET/CT successfully detected intramammary lymph nodes with high ^68^Ga-RM2 uptake, contralateral axillary lymph node metastasis, and bone metastasis. These findings highlighted the potential of ^68^Ga-RM2 PET/CT as a promising imaging modality for the diagnosis of ER-positive BC [78].

The GRPR antagonist radioligand ^67/68^Ga/^111^In/^177^Lu-NeoBOMB1 has demonstrated favorable diagnostic characteristics in preclinical PC models, with ^68^Ga-NeoBOMB1 being particularly effective in detecting PC lesions in patients. To further investigate the diagnostic potential of NeoBOMB1 in GRPR-positive BC, Aikaterini Kaloudi et al. conducted a study using ^67^Ga-NeoBOMB1 in a BC model. They examined the distribution of ^67^Ga-NeoBOMB1, serving as a substitute for ^68^Ga-NeoBOMB1, in GRPR-expressing T-47D cells and animal models. They found that both NeoBOMB1 and ^nat^Ga-NeoBOMB1 exhibited high affinity for GRPR. ^67^Ga-NeoBOMB1 demonstrated strong binding to the cell membrane of T-47D cells but displayed limited internalization, behaving like a radioligand antagonist. Importantly, ^67^Ga-NeoBOMB1 specifically localized in the tumors of mice bearing T-47D xenograft tumors. These findings indicated that ^67^Ga-NeoBOMB1 can effectively target GRPR-positive BC in animal models and holds promising potential for future clinical applications [79].

To identify potential candidates for GRPR-based imaging or targeted therapy, Clément Morgat et al. conducted immunohistochemistry screening of invasive BC to assess the presence and intensity of GRPR expression. The study examined tissue samples from 1432 patients with primary breast tumors who underwent surgery at the Bergonié Institute between 2000 and 2005, prior to receiving neoadjuvant therapy. The researchers investigated the correlation between GRPR expression and various clinical, pathological, and biological parameters, as well as its association with the absence of distant metastasis. The findings revealed that among the 1432 tumor cases, the incidence of GRPR overexpression was 75.8%, which was closely linked to a positive ER status. When considering different molecular subtypes of BC, GRPR was highly expressed in 86.2% of luminal A-like tumors, 70.5% of luminal B-like HER2-negative tumors, 82.8% of luminal B-likeB-like HER2-positive tumors, 21.3% of HER2-enriched tumors, and 7.8% of triple-negative tumors. Notably, in cases where GRPR was overexpressed in breast tumors, 94.6% of metastatic lymph nodes also exhibited high levels of GRPR expression. Given the substantial overexpression of GRPR in a significant proportion of ER-positive tumors, the use of radiolabeled GRPR ligands for imaging and treatment holds promising clinical prospects for patients with ER-positive BC [31].

High expression of GRPR and somatostatin receptor 2 (SSTR2) in BC makes them attractive targets for receptor-mediated nuclear imaging and therapy. Simone U. Dalm et al. conducted a study to assess the suitability of these receptors as targets for metastatic BC. The researchers utilized autoradiography to detect ribonucleic acid expression in human peripheral blood and correlated it with radioligand binding. Furthermore, they quantitatively analyzed the mRNA levels of GRPR and SSTR2 in 60 pairs of primary tumors and corresponding metastatic tumors using reverse transcription polymerase chain reaction (RT-PCR). The expression of receptor mRNA was examined in relation to clinicopathological factors, and a comparison was made between primary tumors and their respective metastatic tumors. The study revealed a significant correlation between radioligand binding of GRPR and SSTR and the expression of receptor mRNA in tumor tissue. Notably, high levels of GRPR and SSTR2 were observed in ER-positive tumors, which aligns with their previous findings and the results reported by Kumar et al. [80, 81]. In Kumar's clinical study, the successful imaging of BC lesions using the GRPR radioligand ^68^Ga-RM2 was positively correlated with ER and PR status. Additionally, Prignon et al. demonstrated that the GRPR agonist ^68^Ga-AMBA was more suitable for monitoring the response to hormone therapy in ER-positive BC models compared to ^18^F-FDG PET. Regarding SSTR2 expression, there were no significant differences in most cases, except for a notably lower expression of the SSTR2 gene in liver and ovarian metastatic tumors compared to corresponding primary tumors [82]. Consequently, nuclear imaging and/or targeted therapy to GRPR and SSTR2 hold promise for improving the care of both primary and metastatic BC [83].

Zhang et al. conducted a study on the potential application of the dual-targeting tracer ^68^Ga-BBN-RGD, which targets both GRPR and integrin α_v_β_3_, in PET/CT imaging of BC and metastatic tumors. The study included 22 female patients with BC, of which 16 were diagnosed with BC using molybdenum target X-ray examination, and the remaining 6 underwent radical mastectomy. All 22 patients underwent PET/CT imaging for 30–45 min following intravenous injection of ^68^Ga-BBN-RGD. Additionally, 11 patients underwent ^68^Ga-BBN PET/CT within 2 weeks. The final diagnosis was based on histopathological examination of surgical resection or biopsy samples. The imaging results demonstrated positive accumulation of ^68^Ga-BBN-RGD in both primary and metastatic lesions. The maximum standardized uptake value (SUV_max_) of ^68^Ga-BBN-RGD PET was found to be higher in the ER-positive group compared to the ER-negative group. Moreover, the mean SUV values of ^68^Ga-BBN-RGD showed a strong correlation with the expression of both GRPR and integrin α_v_β_3_ in both primary and metastatic lesions. This dual-targeting radiotracer exhibited significant uptake in primary and metastatic lesions of BC. ^68^Ga-BBN-RGD PET/CT hold great potential in the differential diagnosis of BC, axillary lymph node metastasis, and distant metastasis [84].

Morgat et al. reviewed and compared the differences between ^68^Ga-RM2 PET and ^18^F-FDG PET in different phenotypic BC. The binding of ^68^Ga-RM2 in GRPR-expressing tumors was significantly higher than that in GRPR-negative tumors (*P* = 0.022). In ER^+^ tumors, the binding of ^68^Ga-RM2 was significantly higher than that of ^18^F-FDG (*P* = 0.015). In tumors with low expression of Ki-67, the binding of ^68^Ga-RM2 was also significantly higher than that of ^18^F-FDG (*P* = 0.029). In general, the binding of ^68^Ga-RM2 and ^18^F-FDG in tumor specimens showed the opposite pattern, and the binding of ^68^Ga-RM2 in low ^18^F-FDG-bound tumors was significantly higher than that in high ^18^F-FDG-bound tumors (*P* = 0.021). This negative correlation was also recorded in a small number of patients who underwent ^18^F-FDG PET/CT before surgery. The results of this study showed that GRPR-targeted radiotracers can act as an alternative in PET/CT imaging of ER^+^ breast tumors [85].

### Other cancers

Mattei et al. conducted an analysis of 238 lung cancer specimens, encompassing both SCLC and NSCLC, and investigated the relation between immunohistochemical results and clinical stage, cell type, sex, and survival rate [33]. The study revealed that GRPR expression was more abundant in advanced stages of the disease, indicating a significant correlation between clinical stage and GRPR expression. While the expression of GRPR is generally similar in SCLC and NSCLC, NSCLC exhibited higher GRPR intensity. Traditional ^18^F-FDG lacks specificity and is difficult to distinguish lung tumors from inflammation. However, radioligands targeting GRPR are more specific and can solve this problem much better. Carroll et al. performed an immunohistochemical study on 50 human colon cancer specimens [86]. They observed high expression of both GRP and GRPR in the majority of cancers (62%), while normal adjacent tissues did not exhibit significant expression. An interesting finding was that the co-expression of these two proteins consistently occurred in highly differentiated tumor regions, but not in poorly differentiated tumor regions, suggesting a close association between GRP/GRPR expression and tumor differentiation. GRPR overexpression on the cell surface has also been observed in various other tumors, including head and neck cancer, renal cancer, and intestinal and bronchial carcinoid [87], although limited clinical studies have been conducted in these areas.

## Conclusion

GRPR is commonly overexpressed in multiple cancer types. Some GRPR ligands exhibit favorable binding ability to the receptor and can effectively facilitate in vivo imaging when combined with radionuclides. The utilization of radionuclide-labeled GRPR ligands holds promise for early detection, clinical diagnosis, and treatment of prostate cancer and breast cancer. This review summurized the advances of GRPR-targeted radiotracers for the diagnosis and treatment of cancers, paving the way for significant transformations in clinical practice.

**Table:** 

Name	Sequence	Binding affinity & binding capacity	Human study	Ref
BBN	pGlu-Gln-Arg-Leu-Gly-Asn-Gln-Trp-Ala-Val-Gly-His-Leu-Met-NH_2_	IC_50_ = 1.36 nM (BBN), 1.8 ± 0.2 nM ([Tyr^4^]BBN), 3.3 ± 0.4 nM ([Lys^3^]-BBN), 20.8 ± 0.3(Aca-BBN(7–14))	No	[56–58]
AMBA	Gly-4-aminobenzoyl-Gln-Trp-Ala-Val-Gly-His-Leu-Met-NH_2_	*K*_d_ = 0.7 ± 0.1 nM (^111^In-DOTA-AMBA)	No	[62, 88]
RM1	Gly-4-aminobenzoyl-H–D-Phe-Gln-Trp-Ala-Val-Gly-His-Sta-Leu-NH_2_	IC_50_ = 14 ± 3.4 nM ( ^nat^In-DOTA-RM1) *K*_d_ = 2.4 ± 0.2 nM (^111^In-DOTA-RM1)	No	[62, 88]
RM2	4-amino-1-carboxymethylpiperidine-D-Phe-Gln-Trp-Ala-Val-Gly-His-Sta-Leu-NH_2_	IC_50_ = 7.7 ± 3.3 nM (RM2), 9.3 ± 3.3 nM (^nat^In-DOTA-RM2), *K*_d_ = 2.9 ± 0.4 nM (^111^In-DOTA-RM2)	Yes	[63, 88]
RM26	D-Phe-Gln-Trp-Ala-Val-Gly-His-Sta-Leu-NH_2_	IC_50_ = 5.5 ± 0.4 nM (^nat^Co-NOTA-PEG_3_-RM26)	Yes	[70, 88]
JMV5132	MPAA-βAla-βAla-[H–D-Phe-Gln-Trp-Ala-Val-Gly-His-Sta-Leu-NH_2_]	IC_50_ = 6.8 nM (JMV5132), 3.0 nM (^nat^Ga-NODA-JMV5132), 10.0 nM (Al^nat^F-NODA-JMV5132)	No	[67]
JMV4168	βAla-βAla-[H–D-Phe-Gln-Trp-Ala-Val-Gly-His-Sta-Leu-NH_2_]	IC_50_ = 13.2 nM (JMV4168), 3.2 nM (^nat^Ga-DOTA-JMV4168)	No	[67]
JMV594	H–D-Phe-Gln-Trp-Ala-Val-GlyHis-Sta-Leu-NH_2_	IC_50_ = 2.2 ± 0.1 nM (JMV594)	No	[66]
SB3	p-aminomethylaniline-diglycolicacid-D-Phe-Gln-Trp-Ala-Val-Gly-His-Leu-NHEt	IC_50_ = 4.6 ± 0.5 nM (SB3), 1.5 ± 0.3 nM (^nat^Ga-DOTA-SB3)	No	[69, 88]

## Data Availability

Not applicable.

## Abbreviations

AMP: Adenosine monophosphate
BBN: Bombesin
BC: Breast cancer
CAS: Caspase
CT: Computed tomography
DAG: Diacylglycerol
EGFR: Epidermal growth factor receptor
ERK: Extracellular regulated protein kinases
ER: Estrogen receptor
ESR1: Estrogen receptor 1
FAK: Focal Adhesion Kinase
18F-FCH: 18F-methylcholine
GPCR: G-protein-coupled receptor
GRP: Gastrin releasing peptide
GRPR: Gastrin realizing peptide receptor
HER2: Human epidermal growth factor receptor-2
ICH: Immunohistochemistry
IC50: Half maximal inhibitory concentration
IP3: Inositol triphosphate
ISUP: International Society of Urological Pathology
MAPK: Mitogen-activated protein kinase
MIP: Representative PET images
MRI: Magnetic Resonance Imaging
mRNA: Messenger RNA
NSCLC: Non-small cell lung cancer
PC: Prostate cancer
PET: Positron emission tomography
PIP2: Phosphatidylinositol-4,5-bisphosphate
PKC: Protein kinase C
PKD: Protein kinase D
PLC: Phospholipase C
PR: Progesterone receptor
pro-GRP: Produce gastrin-releasing peptide precursor
PSMA: Prostate-specific membrane antigen
Rho: Ras homologue
RT-PCR: Reverse Transcription-Polymerase Chain Reaction
SCLC: Small-cell lung carcinoma
SPECT: Single photon emission computed tomography
SSTR2: Somatostatin receptor 2
SUV: Standard uptake value
TKI: Tyrosine kinase inhibitors
